# Enrolment and logistical challenges in TaMoVac 01 Phase I/II HIV trial despite the completion of an earlier (HIVIS-03 trial trial) in Dar es Salaam

**DOI:** 10.1186/1742-4690-9-S2-P153

**Published:** 2012-09-13

**Authors:** M Ngatoluwa, P Munseri, M Janabi, F Mhalu, E Sandstrom, M Bakari

**Affiliations:** 1 Muhimbili University of Health and Allied Sciences, Tanzania, United Republic of; 2 Karolinska Institutet, Sweden

## Background

Participation of sub-Saharan countries in HIV vaccine trials is important in the fight against HIV/AIDS, and has to be sustained by continued trials. Experiences from earlier trials are expected to influence the design and the performance of subsequent trials. Following completion of HIVIS-03 trial, TaMoVac- 01 trial was initiated. We compare enrolment experiences between the two trials.

## Methods

The HIVIS-03 trial, conducted between 2007 - 2010 recruited 60 volunteers from the Police force in Dar es Salaam.

The subsequent TaMoVac- 01 trial has recruited 62 volunteers from the Police and Prisons force, and youths at IDC.

## Results

Enrollment of volunteers into the HIVIS-03 took 12 months while the TaMoVac-01 trial took 13 months.

Screened: enrolled ratio was 3:1for HIVIS-03 trail, while for TaMoVac-01 8:1. Reasons for screen-out in the TaMoVac-01 trial were influence of family, misconception, clinical and laboratory abnormalities.

Recruitment of females was a challenge in the HIVIS-03 trial, but was unnoticed in the TMV-01 trial, this could be due to inclusion of youths. Misconceptions in the Police force remain an obstacle to recruitment despite regular education sessions.

Other challenges were: Poor adherence to schedules, due to competing prioritization of employment requirements; difficulties in communication with volunteers without phones.

Most challenges were addressed through collaboration with the concerned authorities in the respective cohorts.

## Conclusion

Recruitment challenges continued in the TMV-01 trial despite our experiences with HIVIS-03 trial. Enhanced Community engagement and timely action by the researchers is necessary to ensure a smooth conduct of the trials.

